# Commentary on: The optimal Dose of Ribavirin for Chronic Hepatitis C: From Literature Evidence to Clinical Practice

**DOI:** 10.5812/hepatmon.7867

**Published:** 2013-02-28

**Authors:** Mortada El-Shabrawi, Mona Isa

**Affiliations:** 1 Department of Pediatrics, Faculty of Medicine, Cairo University, Cairo, Egypt

**Keywords:** Ribavirin, Hepatitis C, Chronic, Interferons

Dear Editor,

We enjoyed reading the excellent article by Abenavoli and colleagues on the clinical role of Ribavirin (RBV), and particularly the selection and maintenance of the optimal RBV dosing strategy that are required to achieve sustained viral suppression in patients with chronic hepatitis C (CHC) infection. They concluded that contemporary therapy for CHC infection is to deliver doses of both RBV and pegylated interferon-alpha (PEG-IFN) that confer optimal antiviral efficacy for a sufficient time to minimize viral relapse. At the same time, it is important to minimize the impact of side effects that might erode the effectiveness of therapy due to dose reductions below the level of therapeutic efficacy, or because the patient is unable to complete an optimal treatment course (1). The early diagnosis and treatment of CHC infection is still a great worldwide healthcare problem. The prevalence of CHC ranges from 0.1% to 6% in different countries. There are an estimated five million chronic hepatitis C virus (HCV) carriers in Western Europe and four million in the United States (2). The incidence of new symptomatic infection has been estimated to be 1-3 cases per1,000,000 annually, with the actual incidence of new infections clearly being much higher, because the majority of cases are asymptomatic (3). A milestone in CHC therapy was the introduction of the guanosine-analogue RBV which has a broad antiviral spectrum, not yet fully elucidated. RBV can cause a decrease in alanine aminotransferase (ALT) levels, but it has a few effects on the HCV RNA levels. RBV is known as an immunomodulator, inhibiting the viral RNA-polymerase, balancing T-helper (Th) 1 and 2 cell responses, and acting by direct cytoprotection (3). We know also from the data of Weber et al. that RBV acts via the signal transduction pathway like other cytostatic agents (4). Over the past decade, significant improvements have been made in the treatment of CHC, especially with the introduction of combined therapy using both interferon and RBV. The optimal dose and duration of treatment is still a matter of debate and importantly the efficacy of this combined treatment varies with the viral genotype responsible for infection. In general, patients infected with HCV genotypes 2 or 3 more readily achieve a sustained viral response (SVR) than those infected with genotype 1. The introduction of the pegylated version of interferon in the past decade has produced better clinical outcomes in patients infected with viral genotype 1. However, the published literature shows no significant improvement in clinical outcomes in patients infected with genotypes 2 or 3 when they are treated with PEG-IFN as opposed to the non-pegylated, when both were given in combination with RBV (5). Conversely, Zeuzem et al. (6) compared the SVR to treatment with PEG-IFN alpha-2b plus RBV for 24 weeks and found a lower rate of response in genotype 3 than in genotype 2 patients: in this multicentre study, the lower SVR in patients with genotype 3 was attributed to the high levels of steatosis in those patients. In the study by Bressler et al., (7) steatosis was not an independent negative predictor of response to CHC treatment, whereas in other studies, the presence of steatosis was negatively associated with SVR in patients with genotype 1 (8-10). Based on the results by Abenavoli et al. (1), weight-based doses of RBV are advantageous for genotype 1-infected patients, but its success in genotype 2- and 3-infected patients is unknown, particularly for shorter treatment durations. Typically, patients are treated by subcutaneous injection of IFN three times per week in conjunction with a daily oral dose of RBV (15mg/kg/day) for 24 to 48 wk. The therapeutic efficacy is typically measured by the following end points: [1] an SVR defined as no detectable levels of the viral RNA in serum at least 24 wk after the end of treatment; [2] a decrease in ALT to within the normal reference range; and [3] signs of histological improvement as demonstrated typically by a paired liver biopsy performed prior to the initiation of therapy and at 24 wk after the end of therapy. Correlations between serum virus levels, serum ALT levels, and liver pathology, while generally acceptable (11, 12), and have not been definitly established. The primary measure of current therapy virtually shows no detectable levels of HCV in serum at least 24 wk following the end of therapy, defined as the SVR. The SVR correlates well in clinical practice with patient recovery and wellbeing (5). The efficacy of the combination therapy with PEG-IFN-α-2b and RBV in children with HCV infection is comparable to and even higher than that reported in adults. This possibly better viral response in children may be associated with shorter duration of HCV infection, lower frequency of liver fibrosis, and iron overload, lower rate of risky behavior (intravenous drug usage and/or alcohol consumption), and lesser co-morbidities (13-15). Treatment with PEG-IFN-α-2b and RBV is associated with several, and sometimes severe, adverse effects, which may affect the treatment efficacy and the patient’s adherence to the treatment regimen. Side effects of such a combination therapy mainly include fever, headache, fatigue, flu-like syndrome, and hematological disorders such as leucopenia, thrombocytopenia, and anemia. Depression, weight loss, thyroiditis, and other autoimmune diseases such as decreasing in height growth, especially in children, have also been reported to be the side effects of the combination therapy (15, 16). In conclusion, successful antiviral treatment has important results as it reduces the number of infected patients, diminishing the spread of the pathogen, inhibiting hepatitis C progression to cirrhosis and perhaps lowering the risk of hepatocellular carcinoma (3). The combination therapy, RBV (more anti-inflammatory, immunomodulant, immunosuppressive) plus PEG-INF (more antiviral), is the standard of care for patients with CHC and/or extrahepatic manifestations of HCV. We still need more effective antiviral modalities with multiple drug combinations for the treatment of CHC. RBV was the first milestone along this road of multiple therapies and to date it is cornerstone of this therapy.
